# Phosphonium-Based Ionic Liquid Significantly Enhances SERS of Cytochrome *c* on TiO_2_ Nanotube Arrays

**DOI:** 10.1021/acsami.2c05781

**Published:** 2022-06-01

**Authors:** Yihui Dong, Mian Gong, Faiz Ullah Shah, Aatto Laaksonen, Rong An, Xiaoyan Ji

**Affiliations:** † Department of Molecular Chemistry and Materials Science, Weizmann Institute of Science, Rehovot 76100, Israel; ‡ Herbert Gleiter Institute of Nanoscience, Department of Materials Science and Engineering, Nanjing University of Science and Technology, Nanjing 210094, P. R. China; § Chemistry of Interfaces, Luleå University of Technology, Luleå SE-971 87, Sweden; ∥ Energy Engineering, Division of Energy Science, Luleå University of Technology, Luleå 97187, Sweden; ⊥ Department of Materials and Environmental Chemistry, Arrhenius Laboratory, Stockholm University, Stockholm SE-10691, Sweden; # Center of Advanced Research in Bionanoconjugates and Biopolymers, ‘‘Petru Poni” Institute of Macromolecular Chemistry, Iasi 700469, Romania; ¶ State Key Laboratory of Materials-Oriented and Chemical Engineering, Nanjing Tech University, Nanjing 211816, China

**Keywords:** surface-enhanced Raman scattering, ionic liquid, TiO_2_ nanotube array, biodetection, ion dissociation, electrostatic interaction

## Abstract

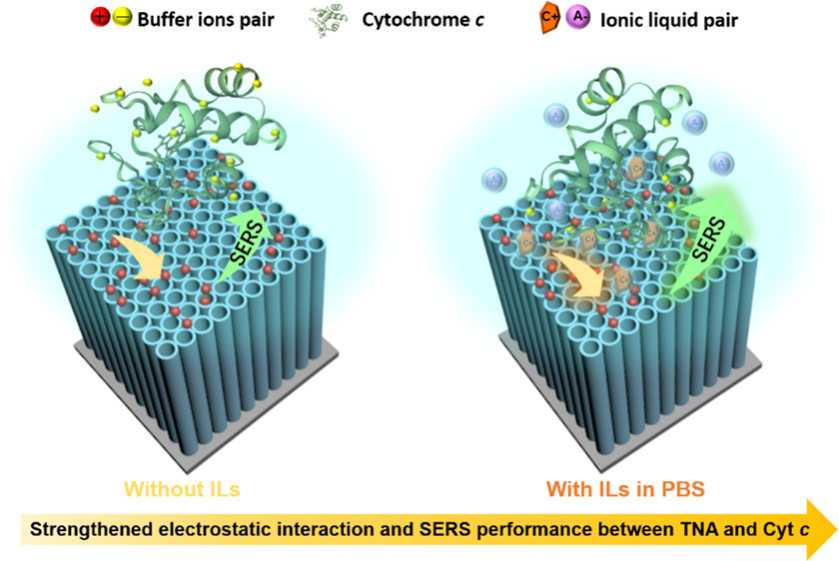

Surface-enhanced Raman scattering (SERS) is an attractive technique for studying trace detection. It is of utmost importance to further improve the performance and understand the underlying mechanisms. An ionic liquid (IL), the anion of which is derived from biomass, [P_6,6,6,14_][FuA] was synthesized and used as a trace additive to improve the SERS performance of cytochrome *c* (Cyt *c*) on TiO_2_ nanotube arrays (TNAs). An increased and better enhancement factor (EF) by four to five times as compared to the system without an IL was obtained, which is better than that from using the choline-based amino acid IL previously reported by us. Dissociation of the ILs improved the ionic conductivity of the system, and the long hydrophobic tails of the [P_6,6,6,14_]^+^ cation contributed to a strong electrostatic interaction between Cyt *c* and the TNA surface, thereby enhancing the SERS performance. Atomic force microscopy did verify strong electrostatic interactions between the Cyt *c* molecules and TNAs after the addition of the IL. This work demonstrates the importance of introducing the phosphonium-based IL to enhance the SERS performance, which will stimulate further development of more effective ILs on SERS detection and other relevant applications in biology.

## 1 Introduction

Biodetection receives increasing attention due to its important role in a variety of biological areas, including disease diagnosis, environmental pollution detection, and food safety.^1^ The pursuit of more sensitive and specific methods for biodetection continues to better understand biological processes.^2^ Many advanced techniques, such as optical microscopy, scanning probe microscopy, and various spectroscopic techniques, have all been applied in biodetections.^3^ As a contribution to developing high-sensitivity spectroscopy techniques, Raman spectroscopy, especially the surface-enhanced Raman scattering (SERS), has been widely used to study the interfacial behavior of biomolecules on solid surfaces.^3−6^

SERS is an ultrasensitive technique and has shown its great potential in high-throughput detection of biomolecules, especially in the detection of proteins.^7^ Compared with other optical detection techniques, for example, surface plasmon resonance, optical waveguide light-mode spectroscopy, and so forth, SERS shows great advantages in terms of noninvasive detection performance and simplicity of operation and sample preparation.^8−11^ However, there are still limitations of the detection performance, especially on the semiconductor-based active-SERS substrates that possess low SERS intensities.^12,13^ To improve the SERS performance, the focus is on adjusting the interfacial properties and interactions, including regulation of active substrates and change in the microenvironment.^6,14^ A wealth of literature has been concentrated on optimizing active substrates, mainly including chemical modifications, doping, and structural regulations.^15−17^ Besides active substrates, changing the microenvironment is another effective way to improve the SERS performance and the detection sensitivity, for example, using additives to adjust the microenvironment, which is critical in regulating the intermolecular interactions and electron-transfer ability.^13,18,19^

Ionic liquids (ILs) are unique materials consisting of organic cations and inorganic/organic anions and having a broad liquid range and their melting points at or below room temperature.^20^ Owing to their unique physicochemical properties, including tunable chemical structures, thermal and chemical stability, high ionic conductivity, and a wide electrochemical window, ILs have rapidly established themselves in a wide range of applications, especially, as additives in bioanalytical chemistry, such as biodetection, drug delivery, and bioextraction,^21−23^ due to their different functions upon adjusting the ion microenvironment.^24^ However, the toxicity of some ILs makes their application challenging, and developing environmentally friendly ILs has become increasingly important.^25^ In biological applications, biocompatibility is an important issue, all leading to high requirements on the biocompatibility and biodegradability for synthesized ILs.^26,27^ Using anionic and cationic counterparts, both derived from natural sources, would be an ideal and sustainable approach to the development of a new generation of ILs, that is, bio-ILs.^27,28^ In our recently published work, the biocompatible choline-based amino acid [Cho][Pro] IL was used as a trace additive in a protein-TiO_2_ system for the first time, allowing us to obtain an increased enhancement factor by three times. Also, the addition of [Cho][Pro] did improve the electron transfer ability.^29^ To study the broad applicability of ILs and to further improve the enhancement on SERS detection, more research on bio-ILs is needed.

The phosphonium-based ILs have been widely used for electrochemical applications due to their advantages in providing higher chemical, thermal, and electrochemical stabilities.^30^ They also showed sufficiently high ionic conductivity, which is promising when used as an environmentally benign electrolyte in electrochemistry.^31,32^ For example, Khan et al. have studied phosphonium-based ILs containing relatively small heterocyclic anions to achieve the desirable properties, including enhanced thermal/electrochemical stability, fast diffusivity of ions, and high ionic conductivities.^33^ Furthermore, for the small heterocyclic anions, 2-furoate anion acid is a good alternative, as it is derived from biomass. It can be conveniently obtained by the oxidation of furaldehyde, typically produced from lignocellulosic biomass.^25,32^

In this work, a phosphonium-based IL with a biocompatible anion was synthesized and used as an additive to modify the microenvironment. Its contribution as an additive to the SERS performance of proteins on the substrates was thereafter studied to clarify the underlying mechanisms in more detail. Phosphonium-based ILs have revealed promising performance in electrochemical applications.^33−35^ Here, we studied an IL comprising the trihexyl(tetradecyl)phosphonium cation ([P_6,6,6,14_]^+^) and the furoate anion ([FuA]^−^), which can be produced on a large scale from renewable sources. For the active substrates, the semiconductors, for example, TiO_2_ nanotube arrays (TNAs), have been recently put at the center of intense research and used as SERS-active substrates due to their high stability, electronic properties, and intrinsically uniform structures.^17,36−39^ Cytochrome *c* (Cyt *c*), a widely used electron-transfer heme protein with a stable charge distribution, was used as a probe molecule for resonance Raman studies.^40^ In this work, two systems were created to study the performance using the IL in the SERS systems and clarify the mechanism, and they are the IL-free system as the reference and the system with the IL in the protein solution. Intrinsically, the SERS enhancement is strongly related to the interactions among the adsorbed molecules, and atomic force microscopy (AFM) is thus used as a powerful tool to detect both adhesion and friction forces for obtaining and verifying the interaction strength to clarify the mechanism at the molecular level.

## 2 Experimental Section

### 2.1 Materials

Cytochrome *c* (Cyt *c*) was purchased from Bio Dee Bio-Tech Co. Ltd (Beijing, China). Sodium bicarbonate and 2-furoic acid (98% purity) were purchased from Sigma-Aldrich. Dichloromethane (Analytical grade) was purchased from Merck. Sodium sulfate anhydrous (VWR chemicals, 99.3% purity) and trihexyl(tetradecyl)phosphonium chloride (SOLVIONIC, >97% purity) were used to synthesize the IL. 16-Mercaptohexadecanoic acid (HS(CH_2_)_15_COOH) was purchased from Sigma-Aldrich Trading Co. Ltd (Shanghai, China). Triethylamine (C_6_H_15_N, 99%), trifluoroacetic anhydride (C_4_F_6_O_3_, 98%), and *N*,*N*-dimethyl formamide (*N*,*N*-DMF, anhydrous) were purchased from J&K Scientific Ltd (Shanghai, China). TNAs were prepared by the electrochemical anodization of titanium foils (Ti, 99%, purchased from Sigma-Aldrich Trading Co. Ltd, Shanghai, China) at an anodization potential of 35 V or 45 V following our previous work.^17^ Deionized water was used in all the experiments.

### 2.2 Synthesis of the IL

**Figure 1:**
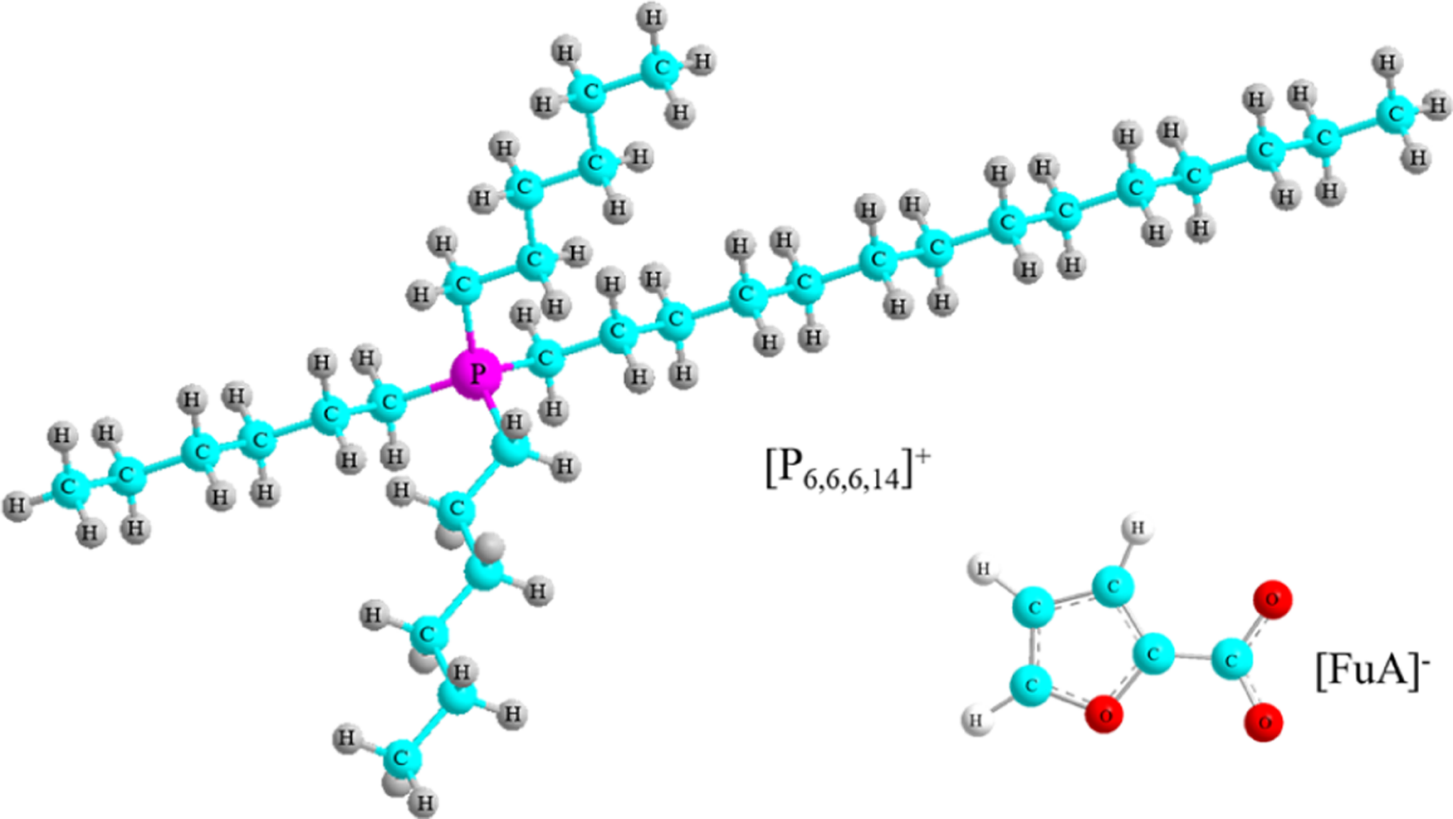
Scheme of the molecular structure of the trihexyl(tetradecyl)phosphonium 2-furoate, [P_6,6,6,14_][FuA].

An aqueous solution of sodium bicarbonate and 2-furoic acid was stirred at room temperature for 3 h. Trihexyltetradecyl-phosphonium chloride was added to the reaction mixture and stirred overnight at 70 °C. The organic layer was extracted with dichloromethane and washed with water three times. The product was dried over sodium sulfate anhydrous and then placed in a vacuum oven at 70 °C for at least 2 days. The structure of the IL (trihexyl(tetradecyl)phosphonium 2-furoate, [P_6,6,6,14_][FuA]) is shown in Figure 1.

### 2.3 Characterization

The structure of the synthesized IL was confirmed using a Bruker Ascend Aeon WB 400 (Bruker BioSpin AG, Fällanden, Switzerland) nuclear magnetic resonance (NMR) spectrometer. The working frequencies were 400.21 MHz for ^1^H, 100.64 MHz for ^13^C, and 162.01 MHz for ^31^P. DMSO-*d*_6_ was used as a solvent, and the data were processed using Bruker Topspin 3.5 software. The NMR resonance lines assignment is given below.

^1^H NMR (400.21 MHz, DMSO-*d*_6_) δ/ppm: 7.45 (1H, s, O–CH=CH), 6.64–6.30 (1H, m, CH–CH=C), 6.34–6.33 (1H, m, CH=CH–CH), 2.22–2.14 (8H, m, 4× PCH_2_), 1.45–1.21 (48H, m, −CH_2_−), 0.86–0.81 (12H, t, −CH_3_). ^13^C NMR (100.63 MHz, DMSO-*d*_6_) δ/ppm: anion (162.72, 154.09, 142.66, 111.72, 111.09), cation (31.92, 30.40, 29.69, 29.59, 22.71, 22.42, 18.37, 17.90, 14.44, 14.38). ^31^P NMR (162.01 MHz, DMSO-*d*_6_) δ/ppm: 33.51. The ^1^H, ^13^C, and ^31^P NMR spectra of [P_6,6,6,14_][FuA] in DMSO-*d*_6_ are shown in the Supporting Information (Figures S1–S3).

Fourier Transform infrared spectroscopy (FT-IR, Nicolet iS10) was used to further characterize the structure of the synthesized IL. Morphology and the surface roughness of TNAs were characterized by field-emission scanning electron microscopy (FESEM, JSM-7800F PRIME) and AFM (Bruker ICON). X-ray photoelectron spectroscopy (XPS, PHI QuantERA II) was used to analyze the percentage of Cyt *c* on TNAs.

### 2.4 Thermal Analysis

The thermal stability of the synthesized IL was assessed through thermogravimetric analysis (TGA) using a PerkinElmer 8000 TGA apparatus. The dynamic TGA experiment was performed with a heating rate of 10 °C min^–1^ under nitrogen gas as the inert carrier gas. 2 mg of the sample was used during the experiment. The onset of decomposition temperatures, *T*_onset_, was calculated from the intersection of the baseline weight and the tangent of the weight versus temperature curve using Pyris software. Differential scanning calorimetry (DSC) was performed using a PerkinElmer DSC 6000 apparatus. About 3 mg of the liquid sample was packed in an aluminum pan. DSC data were recorded during first heating, cooling, and then heating again traces from −80 to 50 °C, with a scanning rate of 5 °C min^–1^. The glass transition temperature, *T*_g_, was determined as the onset of the transition. Inert nitrogen gas was supplied to the instrument with a constant flow of 20 mL min^–1^ to remove air from the sample chamber.

### 2.5 XPS Measurements

X-ray photoelectron spectroscopy (XPS, PHI Quantera II) measurements were performed with a monochromatic Al Kα X-ray source, and the spectra were referenced to the N 1s peak at 399.7 eV, which is owing to the N–C bond in Cyt *c*. Before the XPS measurement, the samples were degassed under high-vacuum conditions to remove the adsorbed water and oxygen.

### 2.6 SERS Measurements

Samples for Raman spectroscopic studies were prepared as follows: the substrates TNA-35 V and TNA-45 V were separately soaked in the 5 × 10^–4^ M Cyt *c* solution (0.01 M PBS solution, pH = 7.2) without ILs and with ILs added into the solution (0.01 g-IL per 20 mL-Cyt *c* solution), respectively, at 4 °C for 12 h. The SERS spectrum was obtained using Raman microscopy (Aramis, Japan) with a 532 nm air-cooled Ar^+^ laser line, and the laser power was controlled at ∼5.4 mW. The typical spectral collection condition was set to be 20 s’ exposure time and two accumulations.

### 2.7 Ionic Conductivity Measurements

The ionic conductivity measurements were conducted with the electrochemical workstation (Questt CS350H) using a two-electrode system. Each TNA was placed in the Cyt *c* solution (5 × 10^–4^ M) with and without IL [P_6,6,6,14_][FuA], respectively, as the working electrode and the other as the reference electrode or auxiliary electrode. The distance between the two electrodes was 2.5 cm. One TNA was connected as during the test, the AC amplitude was set to 20 mV, and the scanning frequency range was 0.01 Hz to 100 KHz. The resistivity was calculated based on the impedance value obtained at 1000 Hz. The inverse of the resistivity represents the ionic conductivities.

### 2.8 AFM Measurements

The measurement of adhesion force was performed with a Dimension Icon AFM instrument in the contact mode at room temperature. The normal spring constant of all the tips was calibrated using the deflection sensitivity of the supported cantilever to transform the normal load signals from volts (V) into the true normal load (N) at the first step. The Cyt *c* molecules were immobilized on the AFM tips coated with gold (XNC12/Cr-Au, MikroMasch) by chemical attachment following our previous work.^41^ During the last step, the tips were immersed into 5 × 10^–4^ M Cyt *c* solution without and with 0.01 g of the IL added into 20 mL of Cyt *c* solution. The tips were washed with the PBS solutions and then dried with N_2_. The adhesion forces were obtained from the force–distance curves at the maximal force jump on retraction, which represents the pull-off force required to separate the tip after contact.^42,43^ About 100 force–distance curves were recorded for analysis.

The friction force measurements were performed in the contact mode with the scan angle at 90° of the tips to the cantilever’s long axis to obtain the lateral force images. The friction forces were derived from the trace and retrace tracks of lateral force images (2 × 2 μm^2^) and given as an output voltage (V) and then transformed into the friction forces (N) according to the torsion of the cantilever.^44^

## 3 Results and Discussion

The work is organized in five parts. The characterizations of the IL [P_6,6,6,14_][FuA] and TNA substrates were carried out in the first part. In the second part, the SERS performance of Cyt *c* on TNAs both with and without ILs was provided to illustrate the advantages of introducing the IL in improving the SERS performance. In the third part, the ionic conductivity was studied to clarify the enhancement mechanism. In the fourth part, the EFs of the SERS performance were determined by combining the XPS results. In the last part, AFM-based adhesion and friction forces were studied to further verify the mechanism of the IL effect on the SERS detection.

### 3.1 Characterization

**Figure 2:**
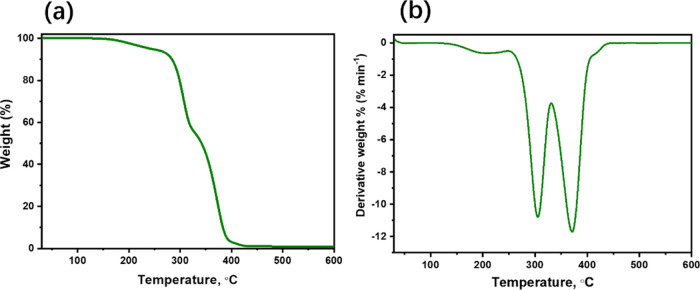
(a) TGA thermogram (b) and DTG curve of [P_6,6,6,14_][FuA] recorded under a nitrogen atmosphere at a heating rate of 10 °C min^–1^.

Figure 2a,b shows the TGA and derivative thermogravimetry (DTG) curves of the [P_6,6,6,14_][FuA] IL at a heating rate of 10 °C min^–1^ under a nitrogen atmosphere. The IL shows weight loss in three steps, indicating that the thermal decomposition takes place in three steps. The first decomposition takes place at 175 °C with about 7% weight loss. The second major decomposition occurs at 289 °C, and totally, 47% weight is lost. Moreover, the high decomposition onset temperature (*T*_d_) of [P_6,6,6,14_][FuA] represents high thermal stability, making it an excellent electrolyte for electrochemical applications.^31^ Finally, the last decomposition step is at 352 °C, as shown in Figure 2a. The DTG curve indicates the weight loss per unit time and at different temperatures, as shown in Figure 2b. The DTG curve shows that the rates of weight loss for the [P_6,6,6,14_][FuA] IL are maximal at ca. 305 and 370 °C, in addition to the slow rate at around 180 °C.

**Figure 3:**
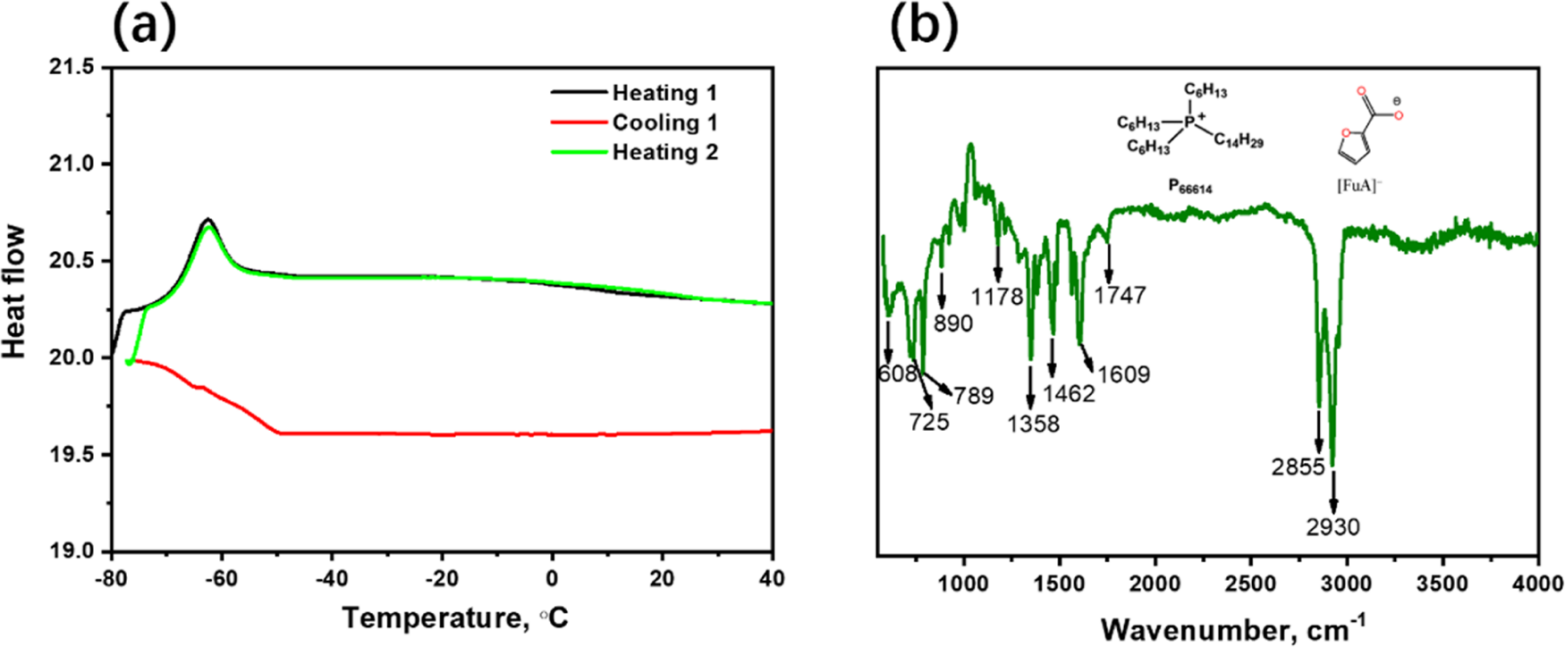
(a) DSC curve of [P_6,6,6,14_][FuA] measured under a nitrogen atmosphere at a heating rate of 5 °C min^–1^; (b) FT-IR of [P_6,6,6,14_][FuA] (inset: molecular structure of [P_6,6,6,14_][FuA]).

The performed DSC experiment as heating–cooling–heating cycles matched very well with the glass transition temperature (*T*_g_) of these two heating cycles (Figure 3a), indicating that the thermal behavior of this IL is reversible. The DSC trace reveals that the [P_6,6,6,14_][FuA] IL is a glass-forming liquid because it has *T*_g_ at the onset of −69 °C. The low *T*_g_ value suggests a low ionic strength and weak cation–anion interactions, and thus, the ions are mobile even at low temperatures. The characteristic bands observed in the FT-IR spectrum of the synthesized IL are shown in Figure 3b. The stretching bands at 2855 and 2930 cm^–1^ are assigned to the sp^2^ and sp^3^ aliphatic C–H of the IL, respectively. The band at around 1747 cm^–1^ is assigned to the C=O of the furoate ring, and the band at around 608 cm^–1^ is attributed to the P–C in the phosphonium cation.^32^ The bending vibrational frequencies at 1462, 1358, and 890 cm^–1^ are assigned to =C–H, −C–H, and C=C functional groups, respectively. At 3400–3100 cm^–1^ representing the −OH groups in furoic acid, no bands were observed in the synthesized sample, confirming the successful synthesis of the IL.^32^

**Figure 4:**
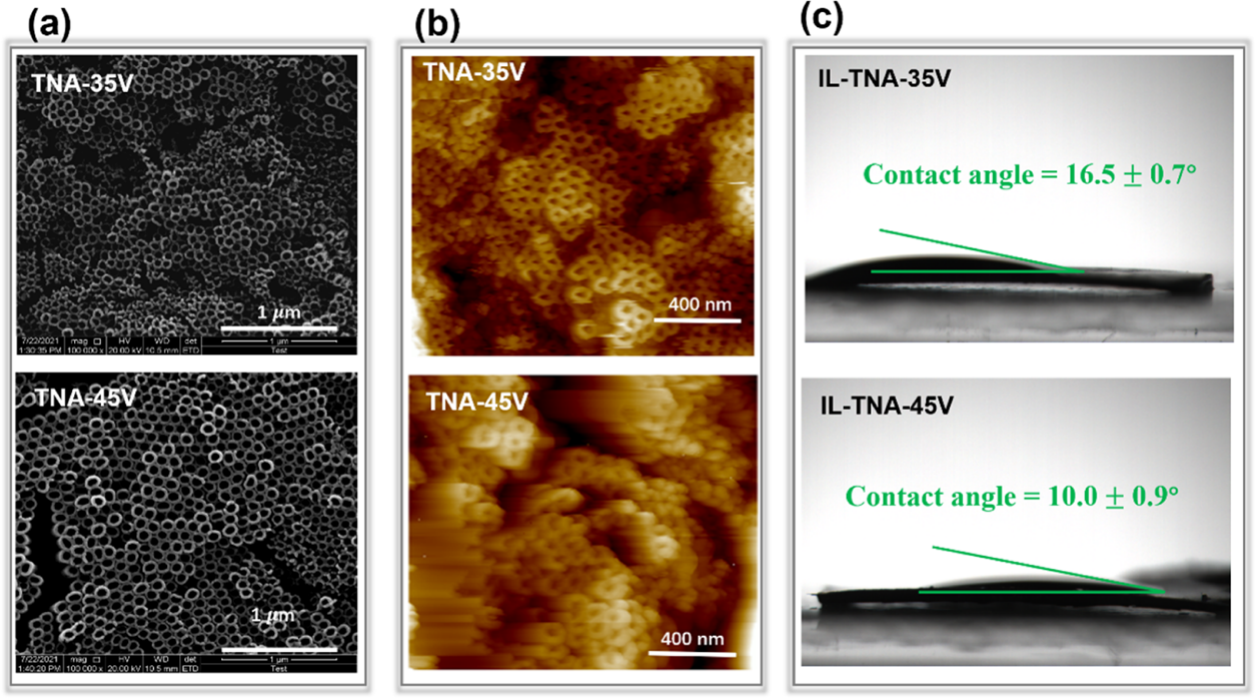
(a) FESEM images and (b) AFM topographic images of TNA-35 V and TNA-45 V; (c) contact angle between [P_6,6,6,14_][FuA] and TNA-35 V/TNA-45 V. The error bar was calculated by measuring at three different regions.

The active substrates used in this work are the TiO_2_ nanotube arrays prepared under anodization voltages of 35 and 45 V, where the tube diameters are 82.4 and 109 nm, and the surface roughness values are 64.3 and 98.1 nm, respectively, as shown in Figure 4a,b. The effective surface areas of these TiO_2_ nanotube arrays were provided by the tube wall depending on the wall thickness. The wall thicknesses of TNA-35 V and TNA-45 V are around 8.8 and 8.0 nm, respectively. By choosing an area of about 500 × 500 nm^2^ from the SEM images in Figure 4a, the ratios of the tube wall area to tube area were estimated to be 49.76:50.24 (∼1:1) and 33.56:66.34 (∼1:2) for TNA-35 V and TNA-45 V, respectively. Additionally, the XRD patterns indicate that the TNA-35 V and TNA-45 V substrates are both in the anatase phase (see Figure S4), excluding the effect of different crystal structures of TNAs on performance.

To verify the hydrophilicity/hydrophobicity of [P_6,6,6,14_][FuA], as well the wettability of [P_6,6,6,14_][FuA] and TNAs, the contact angles between [P_6,6,6,14_][FuA] and TNAs were studied. Here, three different regions were measured to obtain the average values of the contact angle (see Figure S5), and the results were found to be about 16.5 ± 0.7° and 10.0 ± 0.9°, respectively, for the IL on TNA-35 V and TNA-45 V, as shown in Figure 4c. These small contact angles indicate that the interaction between the IL and TNA is strong, and the IL with its excellent hydrophilicity could spread out evenly on these TNA substrates. On the one hand, the smaller contact angle between the IL and TNA-45 V could be due to the larger area occupied by the tube as calculated above, that is, 1:1 and 1:2 for TNA-35 V and TNA-45 V, respectively. On the other hand, the surface roughness of TNA-45 V is larger than that of TNA-35 V, in which a rougher surface provides better wettability.

### 3.2 SERS Performance

**Figure 5:**
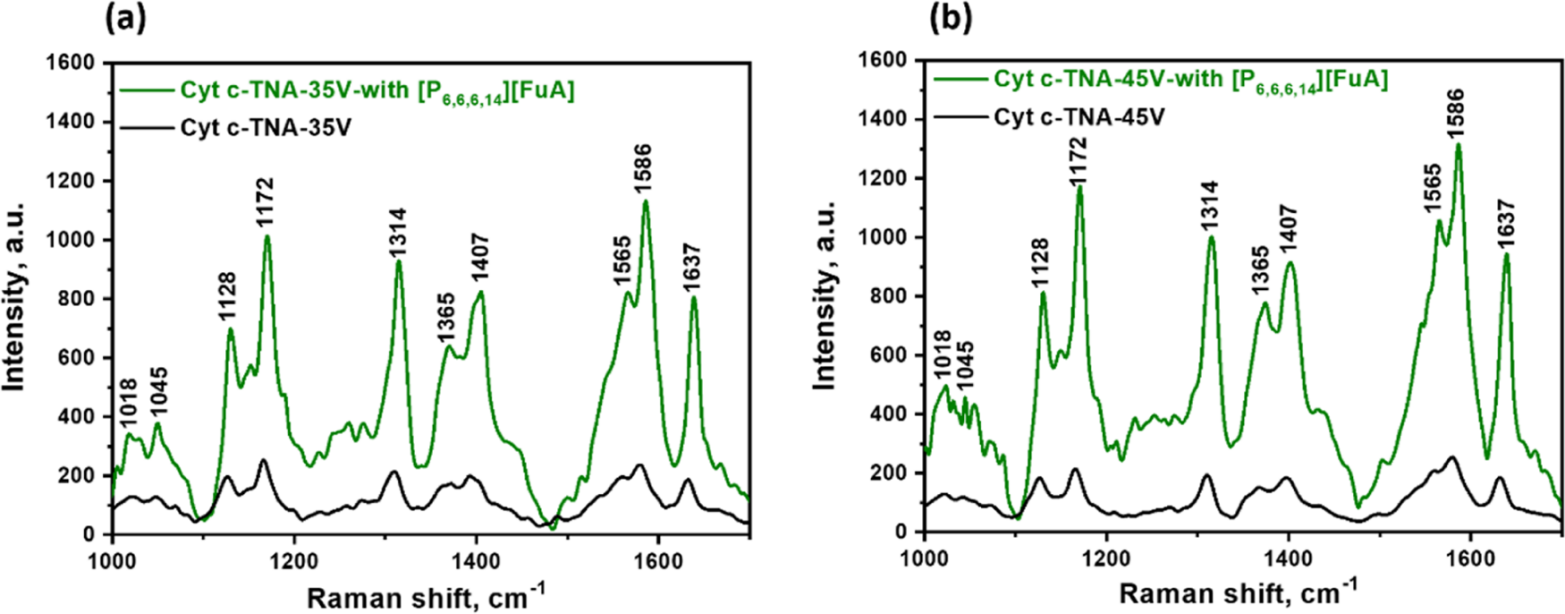
SERS spectra of Cyt *c* on the TNAs prepared under different anodization voltages: (a) 35 V and (b) 45 V, without and with [P_6,6,6,14_][FuA] in the solution.

Based on our previous work, the strongest electrostatic interactions occur between the Cyt *c* molecules and TiO_2_ at pH = 7.2.^17^ Thus, pH = 7.2 was chosen in this work. Since the assigned vibrational modes of Cyt *c* molecules in the 1000–1650 cm^–1^ range are associated with the heme of Cyt *c*, the SERS spectra in the range from 1000 to 1700 cm^–1^ are shown in Figure 5. The detailed normal mode assignment and band locations for the SERS spectra of Cyt *c* adsorbed on the TiO_2_ nanotube array are listed in Table 1. First, based on the assignment and band locations for the Raman spectra of Cyt *c* on TNAs, the characteristic peaks in the spectrum of the ν_4_ (A_1g_) mode at 1364 cm^–1^ and the ν_10_ (B_1g_) mode at 1637 cm^–1^ correspond to the oxidized native states of Cyt *c*, indicating a well-pronounced biological activity of the Cyt *c* molecule on TNAs both with and without an IL. This also evidenced the biocompatibility of [P_6,6,6,14_][FuA].

**Table 1:** Resonance Raman Scattering, Band Locations, and the Normal Mode Assignments for SERS Spectra of Cyt *c*

RRS	mode	symmetry	local coordinate
1018	ν_31_	B_2g_	υ(C_β_–C_1_)_asym_
1045	ν_23_	A_2g_	υ(C_β_–C_1_)_asym_
1128	ν_22_	A_2g_	υ(pry half-ring)_asym_
1172	ν_30_	B_2g_	υ(pry half-ring)_sym_
1314	ν_21_	A_2g_	δ(C_m_–H)
1365	ν_4_	A_1g_	υ(pry half-ring)_sym_
1407	ν_29_	B_2g_	υ(pry quarter-ring)
1565	ν_11_	A_2g_	υ(C_β_–C_β_)
1586	ν_19_	A_2g_	υ(C_a_–C_m_)_asym_
1637	ν_10_	B_1g_	υ(C_a_–C_m_)_asym_

As shown in Figure 5, the intensity of Cyt *c* on TNAs with [P_6,6,6,14_][FuA] is considerably enhanced compared with the reference system without the IL in the Cyt *c* solution, both for the samples of TNA-35 V (Figure 5a) and TNA-45 V (Figure 5b). This strongly indicated that the existence of the IL did enhance the SERS performance. The most intense peaks of Cyt *c* at the substrates with the addition of the IL mainly included the ν_30_ (B_2g_) mode at 1172 cm^–1^, ν_22_ (A_2g_) mode at 1314 cm^–1^, ν_29_ (B_2g_) mode at 1407 cm^–1^, ν_19_ (A_2g_) mode at 1586 cm^–1^, and ν_10_ (B_1g_) mode at 1637 cm^–1^, corresponding to vibrations of the half-ring, C–H, quarter-ring, and C–C. The band locations for the spectra of Cyt *c* adsorbed on TNAs with and without the IL are nearly the same, indicating that the conformations of Cyt *c* at the substrates are consistent with each other for the two studied systems. Meanwhile, the seemingly negligible Raman signal of IL [P_6,6,6,14_][FuA] on the TNA substrate without protein confirmed that the effect of the IL itself on the enhancement of Cyt *c* can be ruled out (see Figure S6).

The enhanced intensity of the peak contains information about the orientation of Cyt *c*. For a heme group of Cyt *c* “lying flat” on the surface, only the symmetric A_1g_ modes are expected to scatter obviously. However, besides the A_1g_ mode, several A_2g_ modes in this system scatter effectively. Especially, the A_2g_ modes at 1314 and 1586 cm^–1^ are nontotally symmetric, evidencing an angle between Cyt *c* and the surface. Furthermore, for the heme group “standing up” on the surface, that is, when the heme in-plane vibrations are mostly perpendicular to the surface, nontotally symmetric B_1g_ modes should exhibit good enhancement. As seen in Figure 5, the B_1g_ mode at 1637 cm^–1^ is enhanced effectively. This indicates that the heme group plane is perpendicular to the surface. Cyt *c* is a membrane electron-transfer protein carrying a +9 charge under the neutral condition, indicating that a strong electrostatic interaction between the heme group and surface exists as the heme group plane is perpendicular to the surface.^40^ The isoelectric point of TiO_2_-related materials is around 5–6, and these TNAs should carry slightly negative charges under the neutral condition (i.e., pH = 7.2 in this study). However, the intensity of Cyt *c* adsorbed on the TNAs without the IL is weak, but it is strong after adding the IL, indicating that the addition of the IL increases the electrostatic interaction between Cyt *c* and TNAs. To verify this, the ionic conductivity was measured to study the chargeability of the TNA-Cyt *c* system with and without the IL. Meanwhile, to verify the stability and reproducibility of the IL-introduced SERS substrates, the measurements of Cyt *c* molecules on these TiO_2_ substrates (TNA-35 V and TNA-45 V, respectively) with and without [P_6,6,6,14_][FuA] were conducted for at least three batches, as shown in the Supporting Information (see Figure S7).

### 3.3 Ionic Conductivities

After adding the IL into the Cyt *c* solution with a large amount of water as the solvent, the IL dissociated either completely or partially into cations and anions due to hydration, and thus, the mixture could be treated as a classical electrolyte solution. This implies that the existence of the dissociated IL could affect the electron transfer ability of the SERS system. Thus, the ionic conductivities (*C*, S/m) of the TNA-Cyt *c* system (taking TNA-45 V as an example) with and without ILs were studied to verify the SERS performances through eq 1
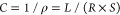
1where ρ, *S*, *L*, and *R* represent the resistivity (m/S), electrode effective area (m^2^), electrode immersion depth (m), and electrical impedance (1/*S*), respectively. The values of the parameters are listed in Table 2, and more details of the calculations are shown in the Supporting Information (Figures S8 and S9).

**Table 2:** Electrical Impedance (*R*, 1/S), Resistivity (ρ, m/S), and Ionic Conductivity (*C*, S/m), of the TNA-Cyt *c* System with and without the IL

sample	*R*, 1/S	ρ, m/S	*C*, S/m
TNA-Cyt *c*	6825	2.76	0.36
TNA-Cyt *c*-IL	4597	1.17	0.85

The resistivity was 2.76 m/S and the ionic conductivity was 0.36 S/m without adding the IL. However, when the IL was added, the resistivity value decreased to 1.17 m/S and the ionic conductivity increased by two to three times to 0.85 S/m. This strongly demonstrates that the existence of IL [P_6,6,6,14_][FuA] can effectively increase the electron transfer ability of the TNA-Cyt *c* system, further leading to an improvement of the SERS performances.

### 3.4 EF Calculation

**Figure 6:**
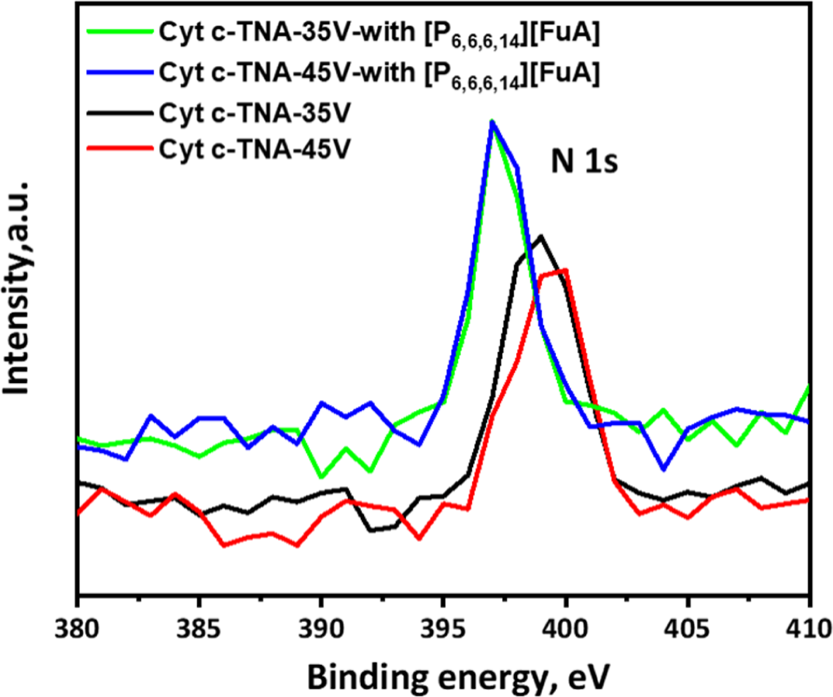
XPS spectra of N 1s for Cyt *c* on TNA-35 V and TNA-45 V with and without IL [P_6,6,6,14_][FuA].

The method to determine the relative enhancement factor EF with respect to the Ti electrode is based on eq 2
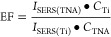
2where *I*_SERS(TNA)_ and *I*_SERS(Ti)_ are the ν_21_ band intensities at 1314 cm^–1^ determined from the SERS spectra and *C*_TNA_ and *C*_Ti_ are the effective adsorption amounts (mol·cm^–2^) of the Cyt *c* molecules on the TNA surface. As the number of adsorbed protein molecules on the small film-like TiO_2_ nanotube surface is extremely low, it is difficult to obtain this value using traditional methods. In this work, we measured the nitrogen content (N 1s) of the Cyt *c* molecules adsorbed on the TNAs surfaces using XPS (see Figure 6). The values of the atomic content of N 1s are listed in Table 3. Meanwhile, the full XPS spectra of the Cyt *c* molecules adsorbed on TNAs with and without the IL are shown in Figures S10 and S11. When the tube diameter is fixed, the ratios of *C*_TNA_ on TNA-35 V and TNA-45 V with and without ILs are 1.19:1 and 1.15:1, respectively. This shows that introducing the IL [P_6,6,6,14_][FuA] could increase the effective amount of adsorbed Cyt *c* molecules.

**Table 3:** Parameters and the Calculated EF in SERS of the TNA-Cyt *c* System with and without the IL [P_6,6,6,14_][FuA]

	nitrogen content, %		*I*_SERS(TNA)_ at 1314 cm^–1^		EF
sample	without IL	with IL	without IL	with IL	ratio, with/without IL
TNA-35 V	11.1	13.2	9.4 × 10^4^	4.5 × 10^5^	4.0:1
TNA-45 V	10.3	11.8	8.9 × 10^4^	4.9 × 10^5^	4.8:1

The effective adsorption amount of Cyt *c* molecules on TNA-35 V is larger than that on TNA-45 V, which is due to the larger area occupied by the tube as calculated above (ratios of the tube wall area and tube area: 49.76:50.24 (∼1:1) and 33.56:66.34 (∼1:2) for TNA-35 V and TNA-45 V, respectively). We have assumed that the *C*_Ti_/*I*_SERS(Ti)_ value was constant, and the values of *I*_SERS(TNA)_ at 1314 cm^–1^ were determined from the SERS spectra, as listed in Table 3. The intensity of *I*_SERS(TNA)_ on TNA-35 V is larger than that on TNA-45 V, which is consistent with the effective adsorption amount. The ratios of the EF on TNA-35 V and TNA-45 V with and without ILs are 4.0:1 and 4.8:1, respectively. This indicates that the introduction of IL [P_6,6,6,14_][FuA] can increase EF by four to five times. Although the effective adsorption amount and the intensity of *I*_SERS(TNA)_ for the Cyt *c* molecules on TNA-35 V without ILs are larger than those on TNA-45 V, the *EF* for the TNA-45 V-Cyt *c* system increases greatly after adding IL [P_6,6,6,14_][FuA]. This indicates that the TNA with a larger pore size and better wettability of ILs possesses excellent performance in enhancing the SERS performance. Also, the surface roughness of TNA-45 V is larger than that of TNA-35 V, which implies that the rougher surface is beneficial for enhancing the SERS performance.^45^ Meanwhile, adding the IL into the system can increase the ionic conductivity by four to five times for the TNA-Cyt *c* system as discussed above, where the increasing order of magnitude is almost consistent with the increase in the EF. This implies a clear correlation between the EF and the ionic conductivity, that is, the electron transfer ability of the TNA-Cyt *c*.

In our previous work, choline-based amino acid [Cho][Pro] was added into the solution, and the EFs were enhanced by two to three times,^29^ which is not as good as that found for the phosphonium-based IL [P_6,6,6,14_][FuA] in this work. This implies that the phosphonium-based IL exhibits a clearly better performance in the SERS detection.

### 3.5 AFM-Based Force Measurements

**Figure 7:**
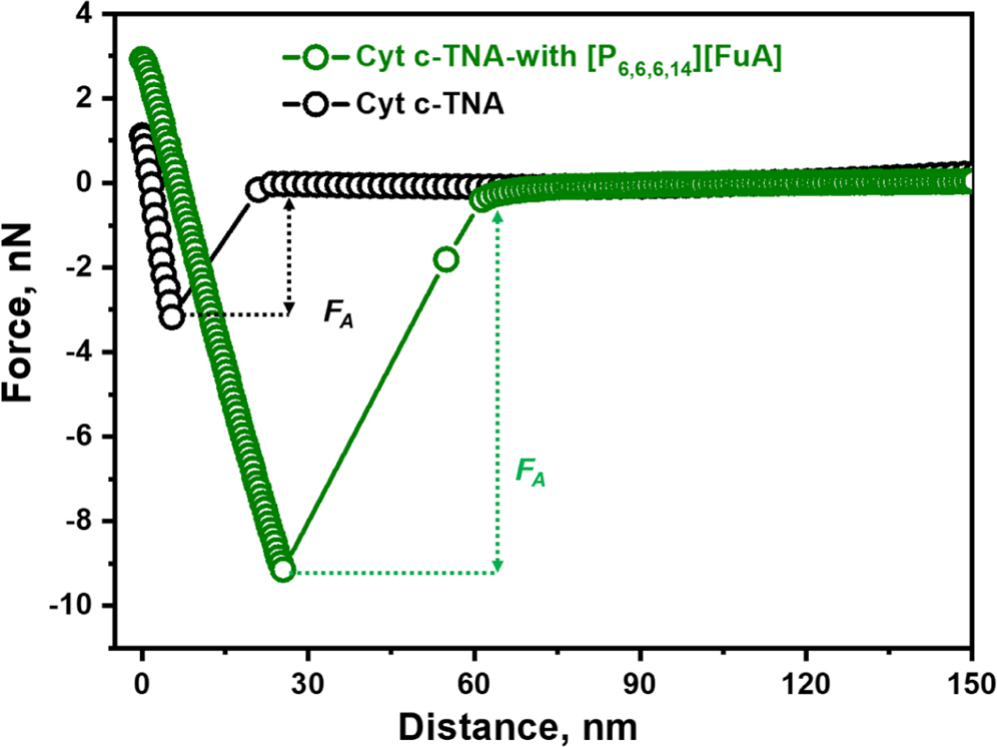
Representative retracting force–distance curves for TNAs with Cyt *c* in the systems with and without IL [P_6,6,6,14_][FuA]. *F*_A_: adhesion force.

The AFM-based adhesion and friction forces were studied to further verify the effects of the hydrated IL on the Cyt *c* interaction with TNAs. Since the surface roughness of TNA-45 V is higher than that of the TNA-35 V, it may interfere with the study of the actual interaction, and TNA-35 V was chosen to further study the Cyt *c* interaction with TNAs with and without the IL in this part. The representative force–distance curves are shown in Figure 7, and the adhesion force (*F*_A_, nN) and friction force (*F*_s_, nN) of Cyt *c* with TNAs with and without the IL are listed in Table 4. It is clear found that the addition of the IL increases *F*_A_, indicating a stronger interaction strength between Cyt *c* and TNAs. This is also consistent with the results that the SERS intensity becomes stronger after adding the IL.

**Table 4:** AFM Measurements of the TNA-Cyt *c* System with and without the IL

sample	*F*_A_, nN	*S*_c_, m^2^	*F*_A_/*S*_c_, nN/m^2^	*F*_s_, nN
Cyt *c*-TNA	3.68 ± 1.81	1.39 × 10^–16^	2.65 × 10^16^	2.79 ± 0.70
Cyt *c*-TNA-with IL	9.80 ± 3.73	2.67 × 10^–16^	3.67 × 10^16^	7.13 ± 2.20

The adhesion force measured by AFM is related to the number of the protein molecules adhered on the tip and the contact area between the protein and surface. To discuss the adhesion force quantitatively, the effective contact area (*S*_c_ in m^2^) between the protein cluster-coated tip and TNA surface was calculated with the Hertz and Johnson–Kendall–Roberts theories.^46^ The results are listed in Table 4. The adhesion force per unit contact area (*F*_n_/*S*_c_ in nN/m^2^) was also obtained (see Table 4). The effective contact area is proportional to the total force, and the difference in *F*_n_/*S*_c_ states a different number of the protein molecules interacting effectively with the substrates.

Meanwhile, the friction force becomes strengthened due to the corresponding stronger interaction forces that would require higher energy to break the adhesion. The friction force was about 2.79 ± 0.70 nN for the system without the IL, whereas for the system with the IL, it is about 7.13 ± 2.20 nN. Therefore, the increase in electrostatic forces led to an increase in the lateral frictional resistance, providing higher friction force. These AFM-based adhesion and friction forces support the effectively enhanced SERS performance for the system with the addition of the IL [P_6,6,6,14_][FuA].

### 3.6 Mechanism Analysis and Discussion

**Figure 8:**
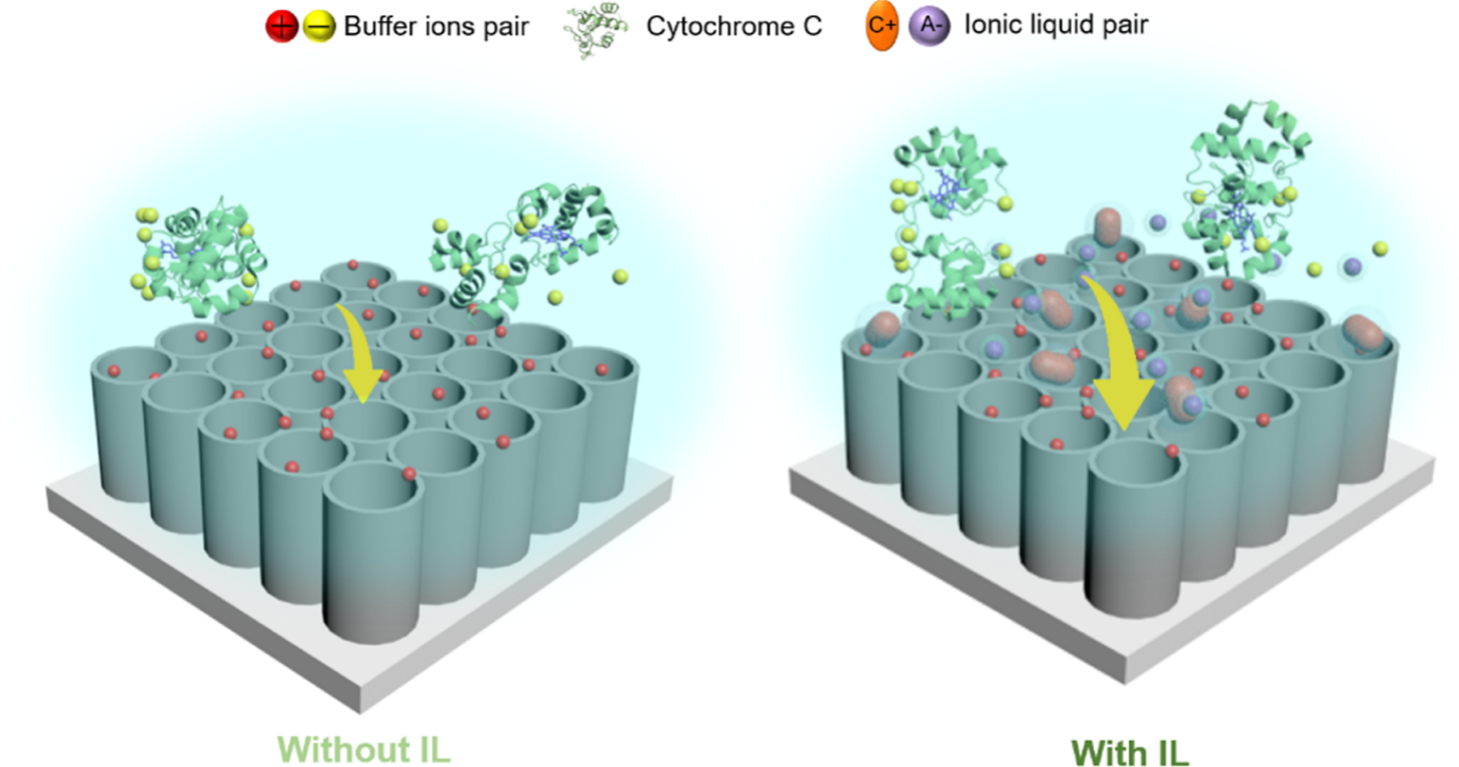
Schematic representation of the different interaction behaviors of Cyt *c* with TNAs in the system without and with the IL in solution. The size of the yellow arrow represents the strength of the interaction force.

According to the SERS results, the heme group plane of Cyt *c* is located perpendicular to the TNA surface with strong electrostatic interactions between the heme group of Cyt *c* and the TNA surface. This indicates that the addition of IL [P_6,6,6,14_][FuA] increases the interaction strength between Cyt *c* and TNAs. Based on our results and observations, as well as the available literature, we can give some suggestions about the possible mechanism that could lead to an enhanced SERS performance. This could be further verified by molecular simulations in the future. We assumed that the IL [P_6,6,6,14_][FuA] becomes dissolved and then dissociates to some degree after adding it into the protein solution. Both the dissociated cations and anions get hydrated by the water molecules. Although, in IL [P_6,6,6,14_][FuA], the [FuA]^−^ anions are relatively small and the charge density distribution is more delocalized due to the aromaticity,^31^ they are still well solvated in water as water can be attracted to the aromatic ring center from both sides of the ring. Despite the [P_6,6,6,14_]^+^ cations having localized charge centers, the positive charge center in the [P_6,6,6,14_]^+^ cation is well shielded by the four long hydrophobic tails, making it difficult for water to approach and hydrate it, decreasing the solvation possibility and degree and leading to the “hydrophobic” nature of the cation.^47,48^ Thus, the “hydrophobic” nature of the [P_6,6,6,14_]^+^ cation will approach TiO_2_, which is slightly negatively charged based on the counterion release mechanism. This further increases the hydrophobicity of the TiO_2_ surface, resulting in a strong electrostatic interaction between Cyt *c* and the TiO_2_ surface (Figure 8), as the hydrophobic surface achieves highly attractive interactions with protein due to the hydrophobic force.^49^ Hence, the addition of IL [P_6,6,6,14_][FuA] effectively increased the electrostatic interaction between Cyt *c* and TNAs, further enhancing the SERS performance.

Moreover, guided by our recently published work, the IL cation with a longer chain length (e.g., [P_6,6,6,14_]^+^) can lead to an increased molecular interaction force between ILs and the charged SiO_2_ surfaces,^50^ and thus, adding [P_6,6,6,14_][FuA] into the Cyt *c*-TNA system strengthens the interaction force between Cyt *c* and TNAs due to the long chain length of the [P_6,6,6,14_]^+^ cation, leading to a better enhanced SERS performance, when compared to the choline-based amino acid [Cho][Pro] with a shorter chain length of the cation.

## 4 Conclusions

The new biocompatible and biodegradable hydrophobic IL [P_6,6,6,14_][FuA], the anion of which is derived from biomass, a renewable source, was used as a trace additive into protein solution to obtain an improved SERS performance of Cyt *c* on the TNA-based substrates. The *EFs* were found to increase by four to five times after adding [P_6,6,6,14_][FuA], verifying the increased electron-transfer ability of the SERS system. The ionic conductivity of the system increased due to the dissociation of the IL, and the long chain of the [P_6,6,6,14_]^+^ cation led to a stronger interaction of Cyt *c* with TNAs. The AFM-based adhesion and friction forces further verified the strong electrostatic interaction between the Cyt *c* molecules and TNAs with the addition of the IL. Based on our findings, the introduction of IL [P_6,6,6,14_][FuA] demonstrated a great improvement in adjusting the microenvironment, while showing a truly remarkable enhancement in the SERS performance for trace detection. The proposed method is expected to stimulate further development of new ILs for SERS applications in biology, bioanalysis, and nanoscience to mention a few.

## Supporting Information Available

1H NMR spectrum of [P_6,6,6,14_][FuA]; ^13^C NMR spectrum of [P_6,6,6,14_][FuA]; ^31^P NMR spectrum of [P_6,6,6,14_][FuA]; XRD patterns of TNAs; contact angles between [P_6,6,6,14_][FuA] and TNAs; Raman signal of [P_6,6,6,14_][FuA] on TNA; repetitive SERS measurements of Cyt *c* on TNAs; ionic conductivity measurement without [P_6,6,6,14_][FuA]; ionic conductivity measurement with [P_6,6,6,14_][FuA]; full XPS spectra for the Cyt *c* on the TNA-35 V without and with [P_6,6,6,14_][FuA]; and full XPS spectra for the Cyt *c* on the TNA-45 V without and with [P_6,6,6,14_][FuA] (PDF)
