# Supplementary material for: Phosphonium-Based Ionic Liquid Significantly Enhances SERS of Cytochrome c on TiO2 Nanotube Arrays
Source: ACS Appl Mater Interfaces. 2022 Jun 1;14(23):27456–65. doi: 10.1021/acsami.2c05781 (PMC9204693; doi:10.1021/acsami.2c05781)
Supplement: Supplementary file 1 — am2c05781_si_001.pdf [file am2c05781_si_001.pdf]

Supporting information

Phosphonium-based Ionic Liquid Significantly Enhance SERS of Cytochrome *c* on TiO_2 Nanotube Arrays

Yihui Dong[a], Mian Gong[b], Faiz Ullah Shah[c], Aatto Laaksonen[d,e,f,g], Rong An[b]*, and Xiaoyan Ji[d]*

[a]*Department of Molecular Chemistry and Materials Science, Weizmann Institute of Science, Rehovot, 76100, Israel.*

[b]*Herbert Gleiter Institute of Nanoscience, Department of Materials Science and Engineering, Nanjing University of Science and Technology, Nanjing 210094, P.R. China.*

[c]*Chemistry of Interfaces, Luleå University of Technology, SE-971 87 Luleå, Sweden.*

[d]*Energy Engineering, Division of Energy Science, Luleå University of Technology, 97187 Luleå, Sweden.*

[e]*Department of Materials and Environmental Chemistry, Arrhenius Laboratory, Stockholm University, SE-10691 Stockholm, Sweden.*

[f]*Center of Advanced Research in Bionanoconjugates and Biopolymers, ''Petru Poni'' Institute of Macromolecular Chemistry, Iasi 700469, Romania.*

[g]*State Key Laboratory of Materials-Oriented and Chemical Engineering, Nanjing Tech University, Nanjing 211816, China.*

Corresponding Authors

E-mail: ran@njust.edu.cn

E-mail: xiaoyan.ji@ltu.se

1. NMR measurements of [$P_{6,6,6,14}$][FuA]:

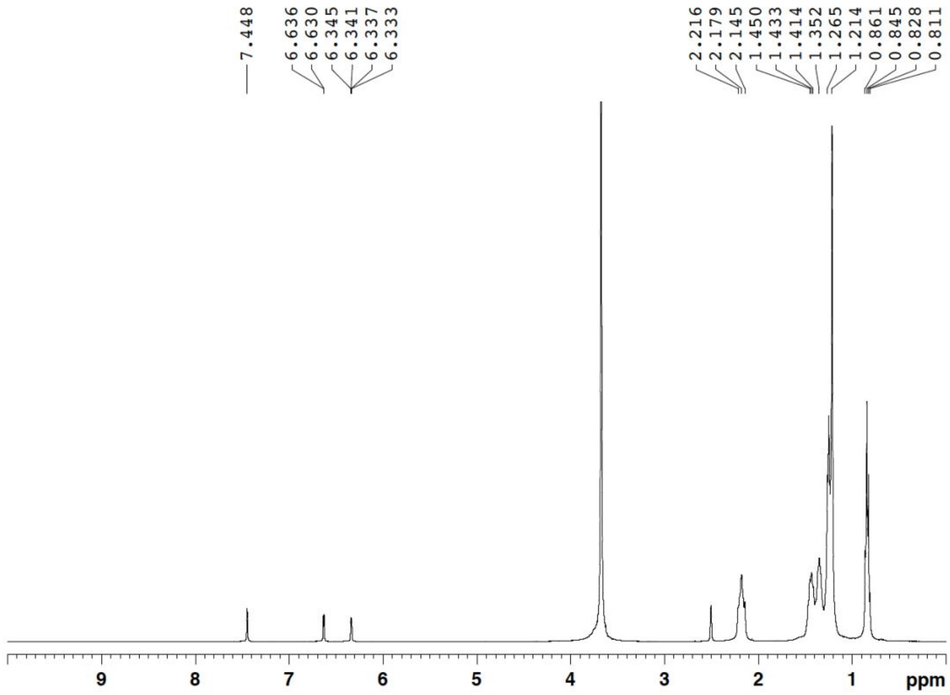

Figure S1. ^{1}H NMR spectrum of [$P_{6,6,6,14}$][FuA] in DMSO-d_6.

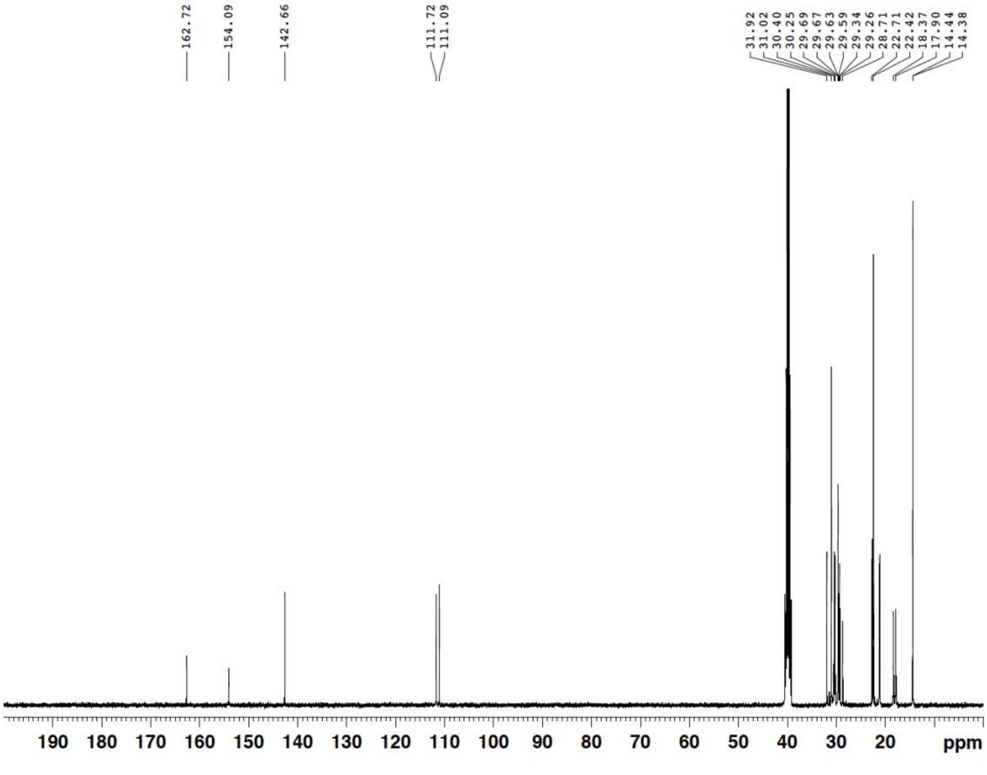

Figure S2. ^{13}C NMR spectrum of $[P_{6,6,6,14}][FuA]$ in DMSO-d_6.

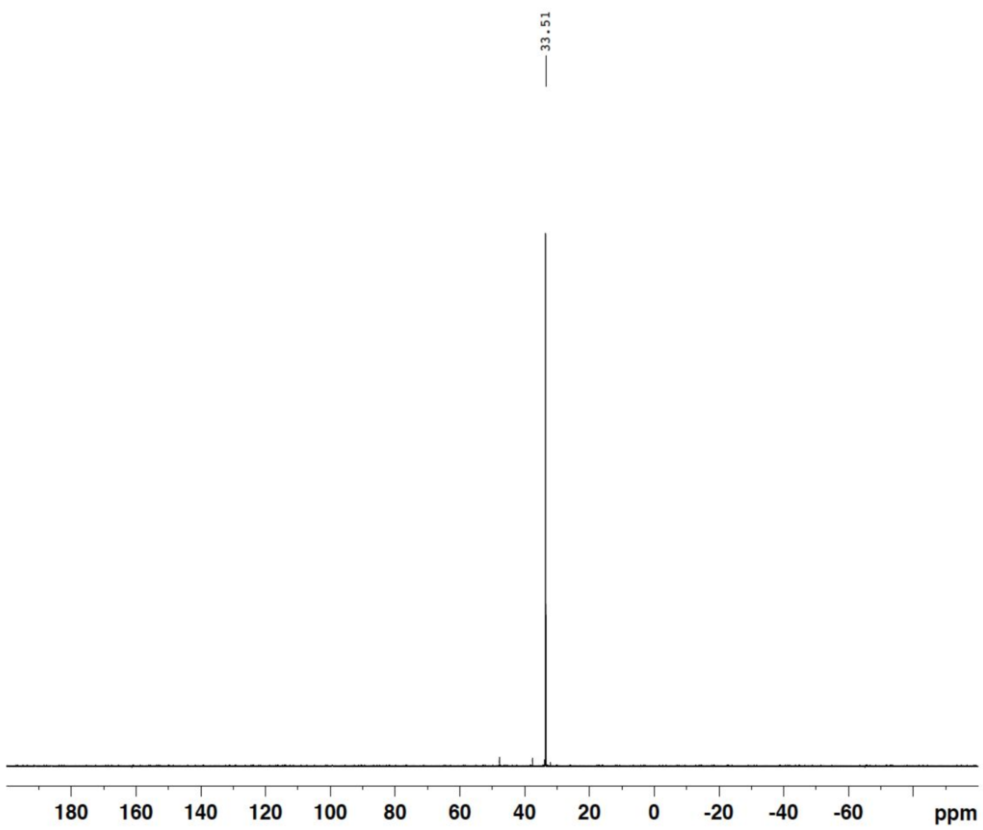

Figure S3. ^{31}P NMR spectrum of $[P_{6,6,6,14}][FuA]$ in DMSO-d_6.

2. XRD measurements of TNA substrates:

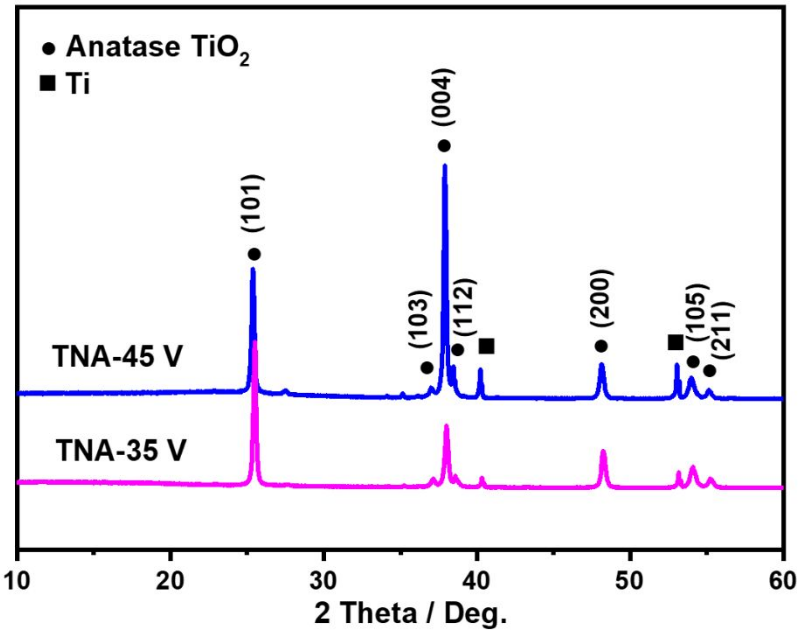

Figure S4. XRD patterns of TNA-35 V and TNA-45 V.

3. Contact angles of IL on TNAs:

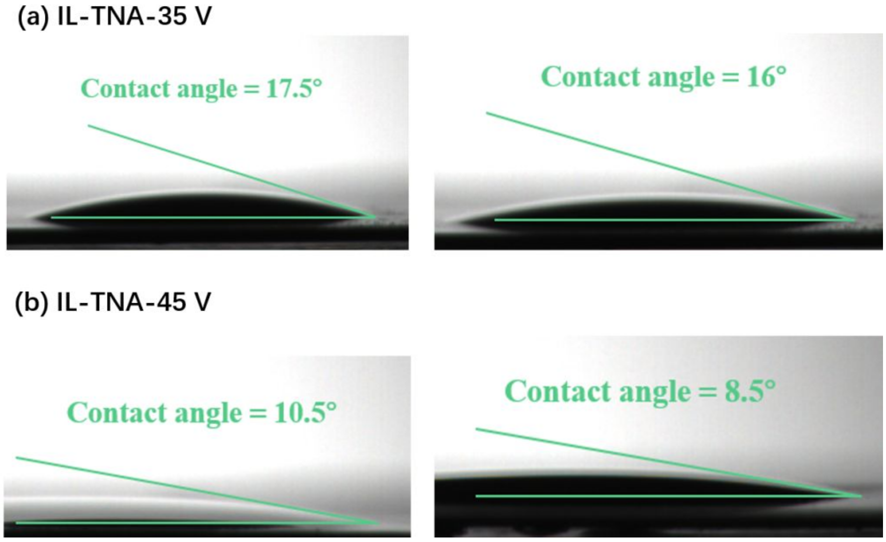

Figure S5. Contact angles between [$P_{6,6,6,14}$][FuA] and TNA-35 V/TNA-45 V at different regions.

4. Raman spectra of IL on TNAs substrate:

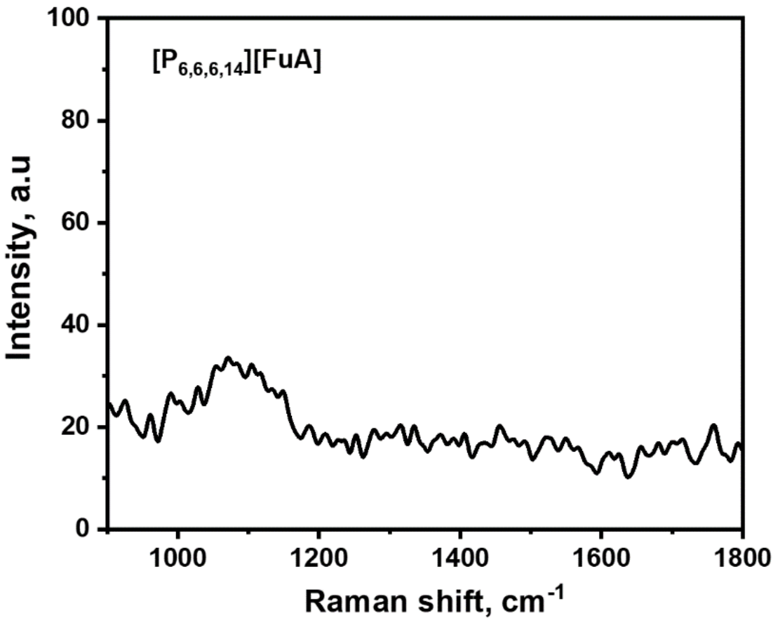

Figure S6. Raman signal of [$P_{6,6,6,14}$][FuA] on the TiO_2 nanotube arrays.

5. Repetitive SERS measurements:

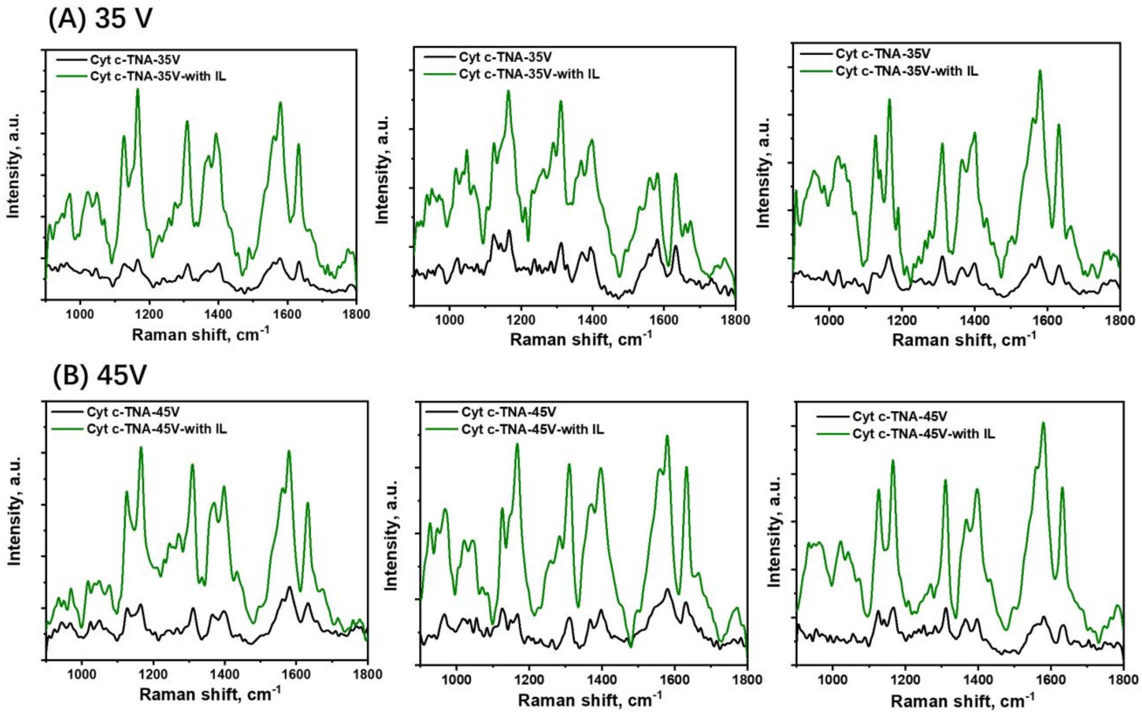

Figure S7. Three batches of the repetitive SERS measurements of Cyt c molecules on (A) TNA-35 V and (B) TNA-45 V with and without [P $_{6,6,6,14}$][FuA], respectively.

6. Ionic conductivity measurements:

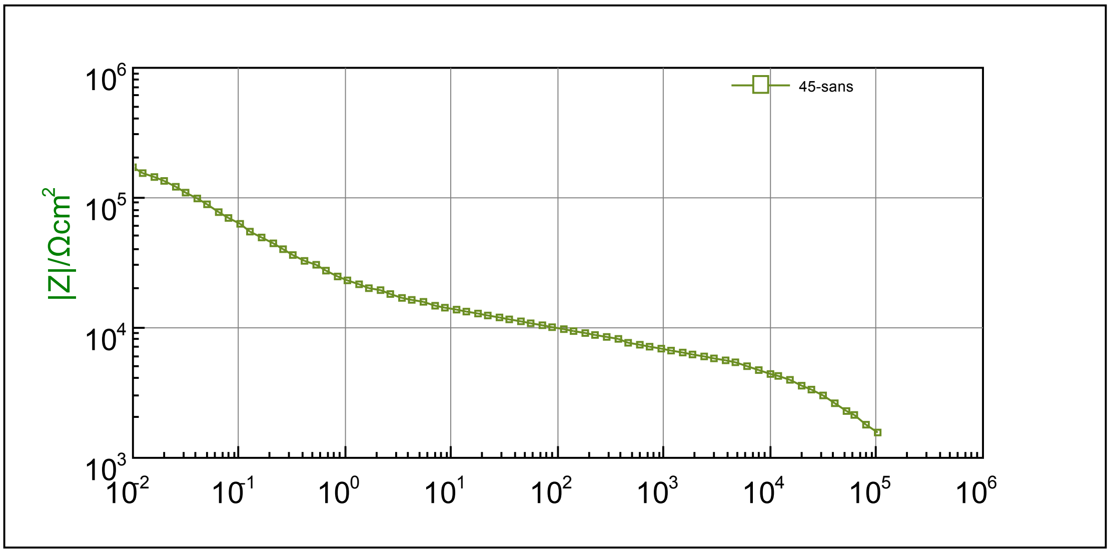

Figure S8. Ionic conductivity measurement of Cyt c on TNA-45 V without [P$_{6,6,6,14}$][FuA].

The ionic conductivities (C, S/m) of the TNA-Cyt c system with and without ILs were

studied to verify the SERS performances above, through the eq. 1 as follow:

$$C=1/\rho = L/(R \times S)$$

Where ρ, S, L and R represent the resistivity (m/S), electrode effective area (m^2), electrode immersion depth (m), and electrical impedance (1/S), respectively.

The value of S is (8.5 $*10^{-3}$)*(0.2*10^{-3}) m^2, L is 4.2*10^{-3} m, R is 6825 which is obtained at 1000 Hz in Figure S4.

$$\rho=\frac{\mathbf{6825} * (\mathbf{8.5} * \mathbf{10}^{-3}) * (\mathbf{0.2} * \mathbf{10}^{-3})}{\mathbf{4.2} * \mathbf{10}^{-3}}=2.76$$

Thus, C= 1/ρ = 0.36 S/m

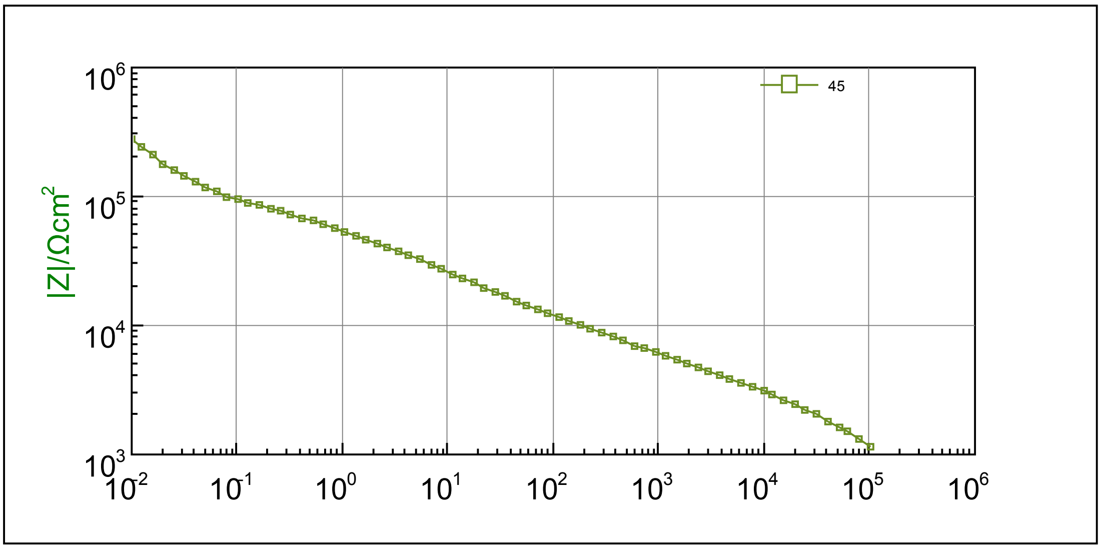

Figure S9. Ionic conductivity measurement of Cyt c on TNA-45 V with adding $[P_{6,6,6,14}]$[FuA].

The value of S is (75 $*10^{-3}$)*(0.2*10^{-3}) m^2, L is 5.5*10^{-3} m, R is 4597 which is obtained at 1000 Hz in Figure S5.

$$\rho=\frac{4597 * (7 * 10^{-3}) * (0.2 * 10^{-3})}{5.5 * 10^{-3}}=1.17$$

Thus, C= 1/ρ = 0.85 S/m

7. XPS measurements:

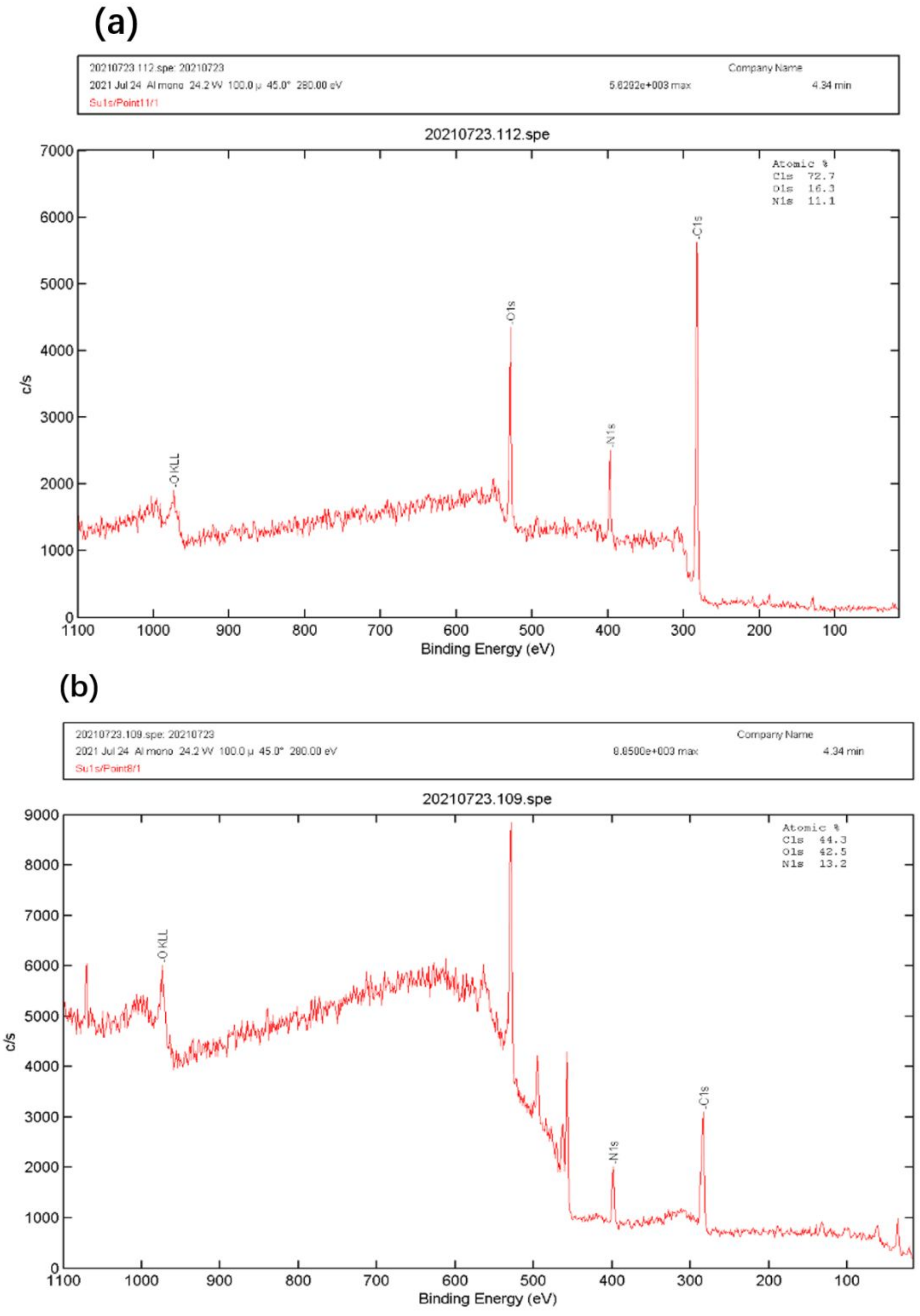

Figure S10. XPS spectra for the Cyt *c* on the TNA-35 V (a)without and (b) with adding [$P_{6,6,6,14}$][FuA].

(a)

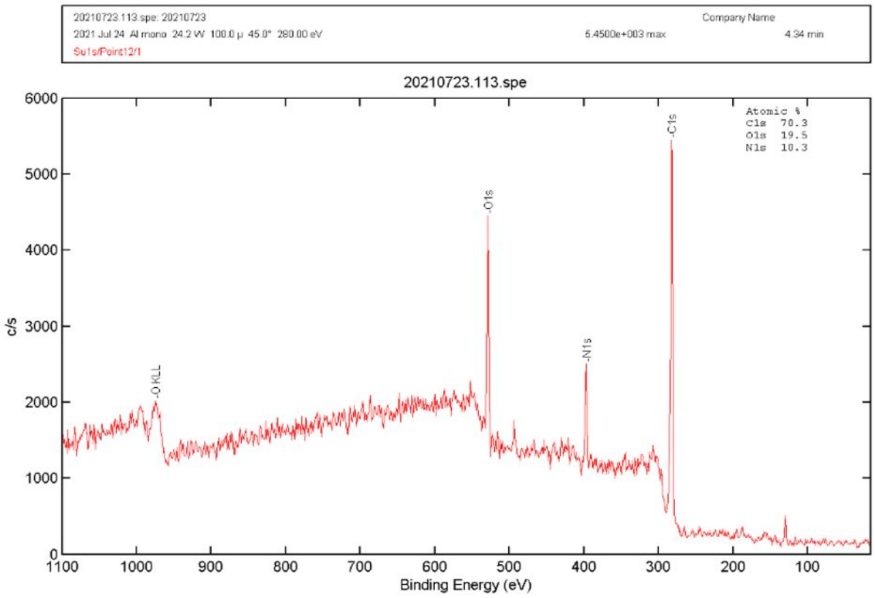

(b)

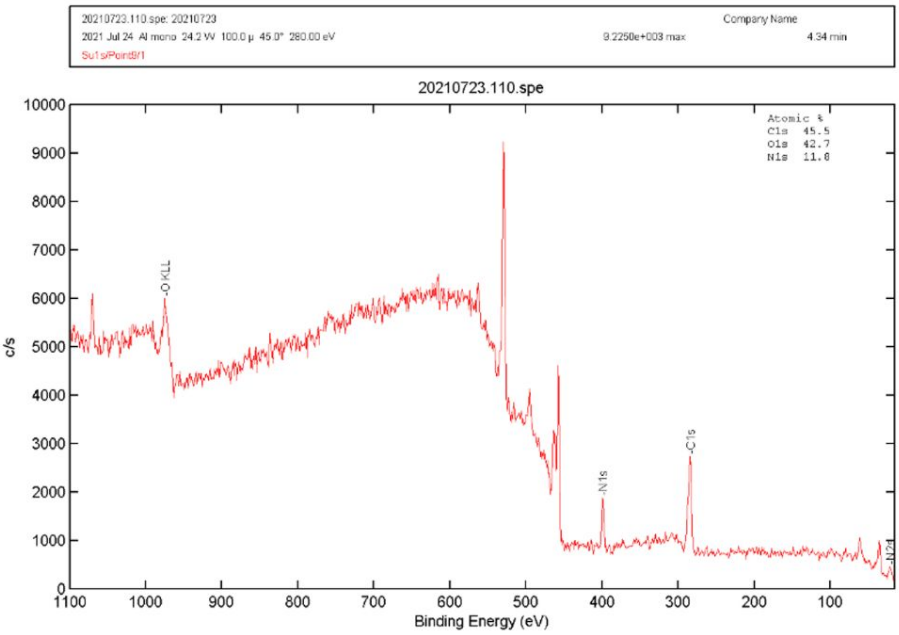

Figure S11. XPS spectra for the Cyt *c* on the TNA-45 V (a)without and (b) with adding

$[P_{6,6,6,14}][FuA]$.
